# Neuroprotective Effect of miR-483-5p Against Cardiac Arrest-Induced Mitochondrial Dysfunction Mediated Through the TNFSF8/AMPK/JNK Signaling Pathway

**DOI:** 10.1007/s10571-022-01296-3

**Published:** 2022-10-20

**Authors:** Qiang Zhang, Haohong Zhan, Cong Liu, Chenyu Zhang, Hongyan Wei, Bo Li, Dawang Zhou, Yuanzheng Lu, Shaomin Huang, Jingge Cheng, Shuhao Li, Chuyue Wang, Chunlin Hu, Xiaoxing Liao

**Affiliations:** 1 Department of Emergency Medicine, The Seventh Affiliated Hospital, Sun Yat-sen University, Shenzhen, 518107 China; 2 Department of Emergency Medicine, The First Affiliated Hospital, Sun Yat-sen University, Guangzhou, 510080 China; 3 National Health Council (NHC) Key Laboratory of Assisted Circulation, Guangzhou, 510080 China

**Keywords:** Cardiac arrest, miR-483-5p, Mitochondrial biogenesis, Oxidative stress

## Abstract

Substantial morbidity and mortality are associated with postcardiac arrest brain injury (PCABI). MicroRNAs(miRNAs) are essential regulators of neuronal metabolism processes and have been shown to contribute to alleviated neurological injury after cardiac arrest. In this study, we identified miRNAs related to the prognosis of patients with neurological dysfunction after cardiopulmonary resuscitation based on data obtained from the Gene Expression Omnibus (GEO) database. Then, we explored the effects of miR-483-5p on mitochondrial biogenesis, mitochondrial-dependent apoptosis, and oxidative stress levels after ischemia‒reperfusion injury in vitro and in vivo. MiR-483-5p was downregulated in PC12 cells and hippocampal samples compared with that in normal group cells and hippocampi. Overexpression of miR-483-5p increased the viability of PC12 cells after ischemia‒reperfusion injury and reduced the proportion of dead cells. A western blot analysis showed that miR-483-5p increased the protein expression of PCG-1, NRF1, and TFAM and reduced the protein expression of Bax and cleaved caspase 3, inhibiting the release of cytochrome c from mitochondria and alleviating oxidative stress injury by inhibiting the production of ROS and reducing MDA activity. We confirmed that miR-483-5p targeted TNFSF8 to regulate the AMPK/JNK pathway, thereby playing a neuroprotective role after cardiopulmonary resuscitation. Hence, this study provides further insights into strategies for inhibiting neurological impairment after cardiopulmonary resuscitation and suggests a potential therapeutic target for PCABI.

**Supplementary Information:**

The online version contains supplementary material available at 10.1007/s10571-022-01296-3.

## Introduction

The consequences of cardiac arrest (CA), including brain injury after resuscitation, are often fatal. It has been reported that only 3–7% of recovering re-establish the neurological state they exhibited before cardiac arrest (Lilja et al. 2015). There is a need to improve neurological outcomes and the quality of life for cardiac arrest survivors as more people survive cardiac arrest. Neuronal mitochondria play central roles in the pathophysiological processes of cardiopulmonary resuscitation (Lou et al. 2021). Ischemia–reperfusion injury is primarily induced by mitochondrial reactive oxygen species (ROS) generated after cardiac arrest. Inhibiting mitochondrial ROS levels reduced neuronal myocardial oxidative stress after the return of spontaneous circulation (ROSC) (Jeggo et al. 1985; Wang et al. 2021). Mitochondria can also control cell death via the induction of apoptosis by releasing cytochrome c after injury (Nhu et al. 2021). Adaptive responses play a role in maintaining mitochondrial function when neurons are exposed to oxygen and glucose deprivation and reoxygenation (OGD/R). These strategies include mitochondrial fission and fusion, mitophagy, and mitochondrial biogenesis, which restore neurovascular homeostasis (An et al. 2021). Therefore, preventing mitochondrial injury after cardiopulmonary resuscitation may be an important strategy for reducing neurological damage after cardiopulmonary resuscitation.

MiRNAs are short nonprotein-coding RNA molecules that are evolutionarily conserved and ubiquitously expressed. Numerous studies have reviewed the role of miRNAs in regulating neuronal apoptosis, regeneration, the plasticity of neurons, and inflammation after cardiac arrest (Roitbak 2020). Due to the stability of miRNAs in the bloodstream and their function in regulating neurological impairment after ischemia‒reperfusion injury, miRNAs have been the most promising newly discovered biomarkers and therapeutic targets of postcardiac arrest that may be used to alleviate neurological impairment (Stammet 2017). In this work, we found that the level of miR-483-5p was correlated with the prognosis of neurological dysfunction through Gene Expression Omnibus (GEO) database screening. However, it is unclear whether miR-483-5p affects the fate of nerve cells after cardiopulmonary resuscitation. The literature shows that the abnormal expression of miR-483-5p is associated with the quality control of mitochondria and with nervous system diseases (Nagaraj et al. 2021; Vazquez et al. 2013). Fan et al. found that miR-483-5p determined mitochondrial fission in tongue squamous cell carcinoma by targeting FIS1 (Fan et al. 2015). Additionally, miR-483-5p upregulated peroxisome proliferator-activated receptor gamma (PPARγ) in stromal cells (Wang et al. 2022), and this effect was closely related to mitochondria and neuroplasticity (Cheng et al. 2010).

TNFSF8 (also known as CD30L and CD153) is a member of the tumor necrosis factor receptor superfamily (TNFSF) (Shinoda et al. 2015) and is primarily expressed in immune system cells (Sun et al. 2013). Similar to TNF-a, TNFSF8 is a product of activated monocytes/macrophages and shows classical pleiotropic cytokine activities. Here, our study found that TNFSF8 was also expressed in neurons and it is the target gene of miR-483-5p. The prominent role of TNFSF in neurological disorder has been already demonstrated in different studies, targeting the miRNA-155/TNFSF10 network restrains inflammatory response in the retina of Alzheimer’s disease, TNFSF12 could promote mitochondrial fusion (Burgaletto et al. 2021; Shunkina Skuratovskaia et al. 2021). In summary, the causal mechanisms are unclear even though the potential role for miR-483-5p in nervous system function after ischemia‒reperfusion injury has been found to be related to mitochondrial dynamics.

Mitochondrial biogenesis is part of mitochondrial quality control and is closely related to oxidative stress levels, mitochondrial-dependent apoptosis after ischemia‒reperfusion (I/R) injury. The activation of mitochondrial biogenesis protects neurons from damage (He et al. 2020a, b). Many cell types, tissues, and disease states have been associated with aberrant AMPK/JNK translocation, including oxidative stress, apoptosis, autophagy, and cell death (Dong et al. 2020; Zhang et al. 2020a, b). However, further study is needed to explore the role played by the AMPK/JNK pathway after cardiac arrest, as miR-483-5p may function in regulating mitochondrial quality control (Hayakawa et al. 2016). The present study was focused on the relationship between mitochondrial dysfunction after cardiac arrest and neuroprotective effects conferred by miR-483-5p and the role that the TNFSF8/AMPK/JNK signaling pathway may play in this process.

## Materials and Methods

### Microarray Data Source Identification of mDEGs

The expression profile data in this study were downloaded from the GEO public database (http://www.ncbi.nlm.nih.gov/geo/); GSE74198 and GSE34643 are miRNA datasets related to the prognosis of neurological function after cardiac arrest. The cerebral performance category (CPC) score has been widely used to evaluate the neurological function of patients with craniocerebral injury. In this study, a CPC score 1–2 was considered an indicator of a good neurological function, and a CPC score of 3–5 was considered an indicator of poor neurological function. The datasets were analyzed with the DEq2 package and limma R package in R language (4.0 vision). The miRNAs were differentially expressed in patients with good and poor outcomes in the two datasets. The screening conditions were set as a *P* value < 0.05 and an absolute value of LogFC > 0.7.

### RNA-Seq Analysis and Functional Enrichment Analysis

Using Illumina HiSeqTM 2000, raw reads in fastq format were processed. Clean data were acquired by removing reads containing poly-N in the raw data, adapters, and low-quality sequences. The expression level of mRNA was calculated using RSEM (RNA-Seq by Expectation Maximization) (v1.3.1) and normalized to FPKM (fragments per kilobase per million reads) (Li and Dewey 2011). The sequencing results were stored with the GSE209531 database (https://www.ncbi.nlm.nih.gov/geo/query/acc.cgi?acc=GSE209531). Edge R software was used to analyze differential mRNA expression between the two groups (Robinsonet al. 2010). Differentially expressed mRNAs were identified on the basis of a | log2ratio |> = 1 and *P* value < 0.05. Enrichment analysis of Gene Ontology (GO) categories and Kyoto Encyclopedia of Genes and Genomes (KEGG) pathways were performed using WebGestalt online software (http://www.webgestalt.org). The WebGestalt database was used to annotate and visualize GO terms and KEGG pathways (Zhong et al. 2022). We identified independent predictors of a neurological outcome using binary logistic regression. The sva package with the ComBat function was used for removing batch effects in the two databases. Then, the four selected miRNAs were subjected to logistic regression analysis.

### Cell Culture, OGD/R Model, and Cell Viability

PC12 cells were purchased from iCell Bioscience Inc., Shanghai and cultured in Dulbecco’s modified Eagle’s medium (DMEM, Gibco, Cat. 12100046, Carlsbad, CA, USA) with 10% fetal bovine serum (Gibco, Cat. 10099-141, NY, USA) and 1% penicillin‒streptomycin (Gibco, NY, USA). PC12 cells were inoculated into 6-well plates at a density of 5 × 10^6^ cells per well. To establish an ischemia-like condition, PC12 cells seeded in glucose-free medium were exposed to hypoxic conditions for 4 h with 1% O_2_, 5% CO_2_, and 94% N_2_. Cells were then incubated with normal culture medium and reoxygenated for 6 h according to the methods in a previous study before functional assays were performed (Yang et al. 2020). The miR-NC-mimics, miR-483-5p mimics, miR-NC-inhibitor, miR-483-5p inhibitor, si-TNFSF8, si-USP25, si-ATN1, and si-NC (GenePharma, Shanghai, China) were transfected into PC12 cells using a Lipofectamine 3000 kit (Invitrogen, Cat. 11668-027, CA, USA) when the cell confluence reached 50–60%. After 48 h of transfection, the cells were subjected to oxygen–glucose deprivation/reperfusion (OGD/R). The JNK activity inhibitor d-epigalbacin (MCE, Cat. 84709-25-1, NJ, USA) and doxorubicin (MCE, Cat. HY-15142A, NJ, USA) were used at the indicated concentrations. The viability of the PC12 cells was measured with a CCK-8 kit (Dojindo, Cat. GB707, Kumamoto, Japan). PC12 cells were seeded in 96-well plates and cultured for 24 h. The absorbance (450 nm) value of the solution was assessed with a microplate reader (BioTek, Winooski, USA).

### Animal Care and Treatment Groups

This research was approved and supervised by the Institutional Animal Care and Use Committee of Sun Yat-sen University (Approval number: SYSU-IACU-2022-001232). As progesterone is a neuroprotective steroid, it reduces proinflammatory cytokine expression and improves neurological outcomes after cardiac arrest (Espinosa-Garcia et al. 2020). In order to eliminate the interference of progesterone, sixty male adult Wistar rats (350–400 g) were obtained from the Animal Experimental Center of Southern Medical University. Food and water were available ad libitum, and three animals were housed in each cage. The animals were randomly divided into five groups: sham group, CA+miR-NC group, CA+miR-Sponge NC group, CA+miR-483-5p group, and CA+Sponge miR-483-5p group. According to the manufacturer’s instructions, an adeno-associated virus-9 (AAV-9, Hanheng, Shanghai, China) system was used to enhance or inhibit the expression of miR-483-5p. A nontargeting scrambled negatively matched shRNA was used as the control. After adequate anesthesia with pentobarbital (40 mg/kg), the animals were placed on a stereotaxic apparatus. A total of 10 μL of the virus were injected intraventricularly at a rate of 0.3 μL/min 3 weeks before the establishment of CA models. Lateral ventricle injection localization was performed as followed: bregma: 1.0 mm lateral, 1.5 mm posterior, and 3.1 mm below the dural surface. Four weeks later, the rats were weighed and anesthetized with 4% sodium pentobarbital.

### Cardiac Arrest Model

Our study involved a 10-min asphyxiation-induced cardiac arrest and cardiopulmonary resuscitation model (Geocadin et al. 2002). When the rats reached 350 g, they were anesthetized with 3% pentobarbital (40 mg/kg) prior to intubation. Intravascular catheters (PE50, Smiths Medical, Ashford, UK) were inserted into the right femoral artery and vein for dynamic blood pressure monitoring and drug delivery. After the connection of a cardiac electrical monitor, the rats were given an intravenous injection of vecuronium (MERCK, Cat. 50700-72-6, Darmstadt, Germany, 3 mg/kg). CA was defined as an average arterial pressure < 30 mmHg. After 10 min, the rats were given extracardiac compression (200 times/min) with ventilator-assisted ventilation (tidal volume: 5 mL, 80 times/min). Then, the rats were given an intravenous injection of adrenaline 0.02 mg/kg once every 3 min until the circulation in the rats returned to normal. Return of spontaneous circulation was defined as the rise of the mean arterial pressure (MAP) to at least 60 mmHg for more than 10 min. The ventilator was removed after the rats resumed spontaneous breathing. In the sham group, the rats were treated with pentobarbital (40 mg/kg) and intubated and then, the femoral vein and femoral artery were catheterized.

### Live/Dead Cell Staining

We used a Live/Dead Cell Double Staining Kit (APExBIO, Cat. K2081, USA) to detect the effects of miR-483-5p on PC12 cells. PC12 cells were cultured in six-well plates for 24 h and then transfected with miRNA mimics or miRNA inhibitors. After OGD/R induction, samples were washed twice with phosphate-buffered saline (PBS) and incubated for 20 min in the dark with a 5 μmol mixed staining solution consisting of Calcein AM-PI. They were then washed with PBS three times, and fluorescence microscopy was performed. Red fluorescence represents dead cells, and green fluorescence represents living cells. We counted the dead cells and determined their proportion among all cells.

### Western Blotting and Isolation of Mitochondria

To obtain proteins, PC12 cells and hippocampal samples were collected and homogenized in RIPA lysis buffer (Thermo Fisher, Cat. 89901, IL, USA). The supernatant was collected after centrifugation at 14,000×*g* for 25 min at 4 °C. Protein concentration was quantified using a BCA assay kit (Thermo Fisher, Cat. 23225, IL, USA). Equal amounts of protein (40 μg) were electrophoresed on 8–12% sodium dodecyl sulfate‒polyacrylamide gel electrophoresis (SDS‒PAGE) gels and then transferred to 0.2-μm polyvinylidene difluoride (PVDF) membranes (Millipore, Cat. ISEQ00010, CA, USA), which were blocked with 5% nonfat milk and incubated overnight with primary antibodies at 4 °C. The primary antibodies were as follows: anti-NRF1 (1:1000, Affinity, Cat. AF5298, USA), anti-TFAM (1:1000, Proteintech, Cat. 22586-1-AP, CHINA), anti-PGC1 (1:1000, Abcam, Cat. ab188102, USA), anti-Cytochrome c (1:1000, Abcam, Cat. ab133504, USA), anti-Bcl-2 (1:1000, Abcam, Cat. ab32370, USA), anti-Bax (1:1000, Abcam, Cat. ab32503, USA), anti-Cleaved caspase 3 (1:500, Cell Signaling Technology, Cat. 9661 s, USA), anti-Caspase 3 (1:500, Cell Signaling Technology, Cat. 9662 s, USA), anti-JNK (1:500, Abcam, Cat. ab179461, USA), anti-phospho-JNK (Y223; 1:1000, Abcam, Cat. ab76572, USA), anti-phospho-JNK (1:1000, Abcam, Cat. ab76572, USA), anti-AMPK (1:500, Abcam, Cat. ab32047, USA), anti-phospho-AMPK (S496; 1:1000, Abcam, Cat. ab92701, USA), TNFSF8(1:500,Taiclone,Cat.tcua7558,China), anti-USP25(1:1000, Abcam, Cat. Ab187156, USA), anti-ATN1(1:1000, Affinity, Cat. DF3824, USA), anti-VDAC1 (1:1000, Abcam, Cat. ab154856, USA), and anti-β-actin (1:1000, Abcam, Cat. ab8227, USA). The next day, after incubation with the HRP-conjugated secondary antibody for 1 h at room temperature, the membranes were detected with a chemiluminescence system (GEAI600, MA, USA). Immunoblots were visualized with a BeyoECL Star chemiluminescence reagent kit and quantified by densitometry using ImageJ software. VDAC1 and β-actin were used as the internal controls. Mitochondrial proteins were extracted from tissue using a mitochondria isolation kit (Beyotime, Cat. C3606, Shanghai, China) according to the manufacturer’s instructions.

### Transmission Electron Microscopy (TEM)

Following CA surgery, rats underwent transcardial perfusion with precooled PBS, followed by treatment with 50 mL of 4% paraformaldehyde. Hippocampal tissue was removed, cut into 1-mm^3^ pieces, and maintained in 4% glutaraldehyde to observe the ultrastructure of the mitochondria. Four regions were randomly selected from three slices of each sample and viewed under ×100,000 magnification. The aspect ratio and proportion of individual mitochondrial ridge areas were measured as morphological parameters using ImageJ software (NIH, Bethesda, MD, USA). The aspect ratio was defined as the ratio of the major axes to the minor axes of the analyzed mitochondria. The proportion of an individual mitochondrial ridge area was defined as the ratio of the area of the mitochondrial ridge to the total mitochondrial area.

### Luciferase Reporter Assay

Jefferson software (http://www.jefferson.edu/) was used to predict the possible binding site of miR-340-5p with the TNFSF8 3′UTR. The TNFSF8 wild-type (Wt) and mutant (Mt) 3′-UTR sequences were synthesized and inserted into a psiCHECK2 plasmid to produce a wild-type TNFSF8 (WT-TNFSF8) luciferase reporter plasmid and mutant TNFSF8 (mut-TNFSF8) luciferase reporter plasmid. Subsequently, a miR-483-5p mimic or mimic-NC was cotransfected with the WT-TNFSF8 reporter or Mut-TNFSF8 reporter plasmid using a Lipofectamine 3000 kit (Invitrogen, Cat. 11668-027, CA, USA). After 48 h, the luciferase activity was measured using a dual-luciferase reporter assay kit (Beyotime, Cat. RG088S, Shanghai).

### RNA Extraction, Reverse Transcription, and Quantitative RT‒PCR

Following the manufacturer’s instructions, total RNA from cells and fresh biopsied tissues was extracted with TRIzol reagent (Invitrogen, Cat. 15596108, CA, USA), and the RNA concentration was measured with a Nanodrop 2000 instrument. The RNA was transformed to complementary DNA (cDNA) with a PrimeScript RT Reagent Kit (Takara, Cat. RR047A, Tokyo, Japan). TB Green™ Premix Ex Taq™ II (Tli RNaseH Plus, Takara, Cat. RR820Q, Tokyo, Japan) was used with a CFX96 Real-Time PCR Detection System (Bio–Rad) to evaluate the relative mRNA levels. The 2-ΔΔCt method was employed to measure the relative mRNA expression. The primer sequences were as follows (5′–3′): Tnfsf8, F: GCAATTTCTCGTGCAGTGCTC,R:TGCCTTGAACTCCAGACTCACA; Usp25, F: TTCTTGAAGCCAGCATAGCAG, R: ACGTTCTTCAGCCCAACCG; Atn1, F: CGCAGATCAAACAGGAACCAG, R: GCACGTCCACCACCTTAGGAG; Rab3b, F: GCAGAGCAACTTGGGTTTGA, R: TCTTATCGCAGATGGCGTCC; Caspas6, F: AGGAACTGCCCTAATCTTCAATCA, R: GTATGCGTAAATGTGGTTGCCTT; C1ql1, F: GGTCACCAACCTAGGCAACAAC, R: TTTCCCTCCATCCAGCTTGA; Kdm4e, F: GACAGTAACATTGCCAACAACACC, R: CAGGAGAAGATAGACCCAGAGGAT; Dnajb7, F: GTTTCACATTCCGTAAGGCAGAT, R: AAAGTCTGGCAAACACTGGGTAG; Foxb2, F: CGCTATAGAGAACATCATCGGG, R: TTGACTGGCACCCCGAATG; Eif3h, F: GAAACACCAGTATCAGCAGCGT, R: TTAATCTGGCCTGCAATGAGC; and Fam131a, F: TCTAACCATCCAGGAGATTGCC, R: AATCGTCCGTGGAGTCATCG.

### Neurological Function Score and Water Maze Experiment

A blinded investigator assessed neurological function using established neurological deficit scores (NDSs). The NDSs of the surviving rats were evaluated 24 h after CA. We evaluated the degree of arousal, cranial nerve reflexes, motor assessment, sensory assessment, motor behavior, seizure, and simple behavioral responses and generated the overall NDS. A score of 80 represents normal brain function and 0 represents brain death. Low scores indicated poor performance. Additionally, Morris water maze tests were performed to assess spatial reference memory. The Morris water maze consisted of a black circular pool (160 cm in diameter and 55 cm high) filled with ink-stained water in which a transparent escape platform (diameter 10 cm) above the water surface was placed in one of the quadrants (the target quadrant). The swimming activity data were collected via a digital video camera placed above the pool, and the data were analyzed with a DigiBehave system (Jiliang Software Company, Shanghai, China). The Morris water maze test consisted of probe and visible platform tests. All rats were given five trials daily for seven consecutive days before CA to ensure that they could find the escape platform within 80 s. There was no significant difference in the time in which the rats in each group found the platform before CA. On Day 7 after CA, the probe test was performed to assess spatial memory retention within 80 s.

### TUNEL Staining

An In Situ Cell Death Detection Kit, TMR red (Roche, Cat. 11684817910, Penzberg, Germany) was used according to the manufacturer’s protocol for terminal deoxynucleotidyl transferase-mediated dUTP nick end-labeling (TUNEL) staining. PC12 cells were fixed with 4% paraformaldehyde for 30 min. The TUNEL solution was added and incubated at 37 °C for 1 h. After the solution was removed, and anti-fluorescence attenuating tablets containing DAPI were added to the cells. The rats were euthanized at 24 h after the operation. The paraffin-embedded hippocampal sections were heated at 60 °C, washed in xylene, and rehydrated with a graded series of ethanol and double-distilled water. Sections were placed in a plastic jar containing 200 mL of 0.1-M citrate buffer, and 300-W microwave irradiation was applied for 5 min. After rinsing twice with PBS, the sections were incubated in a dark, humidified chamber with the TUNEL reaction mixture for 1 h at 37 °C. Then, the sections were washed with PBS and incubated for 30 min. After washing, they were stained with 4′-6-diamidino-2-phenylindole (DAPI) for 15 min. The number of neurons in the stratum pyramidale (200 μm in length) within the CA1 region of the hippocampus was analyzed with a fluorescence microscope equipped with the Image-Pro Plus System (Media Cybernetics, USA). The number apoptotic neurons induced by ischemia‒reperfusion was determined by comparing the number of TUNEL-positive cells with the number of DAPI-positive cells.

### HE Staining and Nissl Staining

The morphological changes in the hippocampal CA1 area of the rats were determined via HE staining and Nissl staining. Rats were euthanized 24 h after cardiopulmonary resuscitation. Brain tissue was routinely embedded in paraffin and cut into 10-μm coronal sections. Sections of brain tissues were stained with HE or Nissl for routine histological examination, and the morphological changes were observed with a light microscope (IX81, Olympus, Tokyo, Japan).

### Flow Cytometry Annexin V-FITC-PI Assay

Cultured PC12 cells were washed with PBS and then digested using EDTA-free trypsin after appropriate treatment. Cell suspensions were collected after centrifugation and suspended in 100 μL of 1× buffer. A FITC Annexin V Apoptosis Detection Kit (BD Pharmingen, Cat. C1062M, Shanghai, China) was used to detect the ratio of apoptotic cells to total cells. Propidium iodide and Annexin V-FITC (5 μL of each) were added to the solution and gently mixed. The final suspensions were then poured into flow cytometry tubes for flow cytometry examination.

### Measurement of Mitochondrial Membrane Potential

A JC-1 mitochondrial membrane potential assay kit (Beyotime, Cat. C2003S, Shanghai, China) was used to measure the mitochondrial membrane potential (MMP) according to the manufacturer’s protocol. JC-1 is a cell-permeable fluorescent dye that accumulates within mitochondria in a potential-dependent manner. Images were taken using an EVOS FL Imaging System. Alteration in the ionic equilibrium resulted in mitochondrial depolarization as indicated by a decrease in the red/green fluorescence intensity ratio. The ratio of fluorescence red intensity/green fluorescence intensity was analyzed using ImageJ software, and the value was calculated on the basis of the control group. Changes in mitochondrial transmembrane potential in the hippocampus were analyzed for rats in each group using 50 mg of fresh hippocampal tissues cut into 1-mm^3^ pieces in PBS and subsequently filtered through 200-mesh sieves. The filtrate containing the brain cells was collected and treated with the JC-1 working fluid. After incubation for 15–20 min at 37 °C in a 5% CO_2_ incubator, the hippocampal cells were centrifuged (2000 RPM, 5 min), washed once, and suspended in PBS. We used a flow cytometer (CytoFLEX, Beckman Coulter, CA, US) (Ex = 488 nm; Em = 530 nm) to detect the MMP. Green fluorescence was detected through FITC channel FL-1, and red fluorescence was detected through PI channel FL-2. Apoptosis was indicated by an increase in the green fluorescence intensity/red fluorescence intensity ratio (Y. Chen et al. 2017).

### ROS Determination by Flow Cytometry

Intracellular ROS levels in PC12 cells were detected with the oxygen-free radical sensitive probe DCFH-DA (Beyotime, Cat. S0033S, Shanghai, China). After OGD/R, the culture medium was discarded and basal medium containing 5-μM DCFH-DA was added to the plates and incubated for 30 min and then, the cells were digested with trypsin. The relative fluorescence intensity was measured by flow cytometry (excitation and emission at 488 nm and 530 nm, respectively). Rat brain homogenates were prepared according to a previously reported method (Jiang et al. 2010), diluted with ice-cold Tris-buffered saline (TBS) at a 1:10 ratio, and incubated for 45 min at room temperature with DCFH-DA (5 mM).

### Oxidative Stress Assay

The supernatant of the PC12 cells and hippocampal homogenates were collected to assess the levels of oxidative stress. A lactate dehydrogenase (LDH) Cytotoxicity Detection Kit (Thermo Fisher Scientific, Cat. C20301, IL, USA) was used to determine intracellular LDH levels. In addition, the supernatant and homogenates were used to investigate both malondialdehyde (MDA) content and superoxide dismutase (SOD) activity using a SOD assay kit (Beyotime, Cat. S0101M, Shanghai, China) and Cell MDA assay kit (Beyotime, Cat. S0131S, Shanghai, China), respectively. Hippocampal ROS levels were measured with a Reactive Oxygen Species (ROS) Fluorometric Assay Kit (Beyotime, Cat. S0033S, Shanghai, China) on a microplate reader.

### Statistical Analysis

SPSS 19.0 (SPSS, IL, USA) software was used for data analysis. Figures were drawn using GraphPad Prism 9 software. All the data are expressed as the mean ± SD. A t test was performed to compare two groups, and a one-way analysis of variance (ANOVA) was performed for group of three or more. A *P* value < 0.05 was considered to be statistically significant.

## Results

### High miR-483-5p Expression Was Associated with Neurological Prognosis After Cardiopulmonary Resuscitation and Alleviated Cell Injury After Ischemia‒Reperfusion

**Fig. 1:**
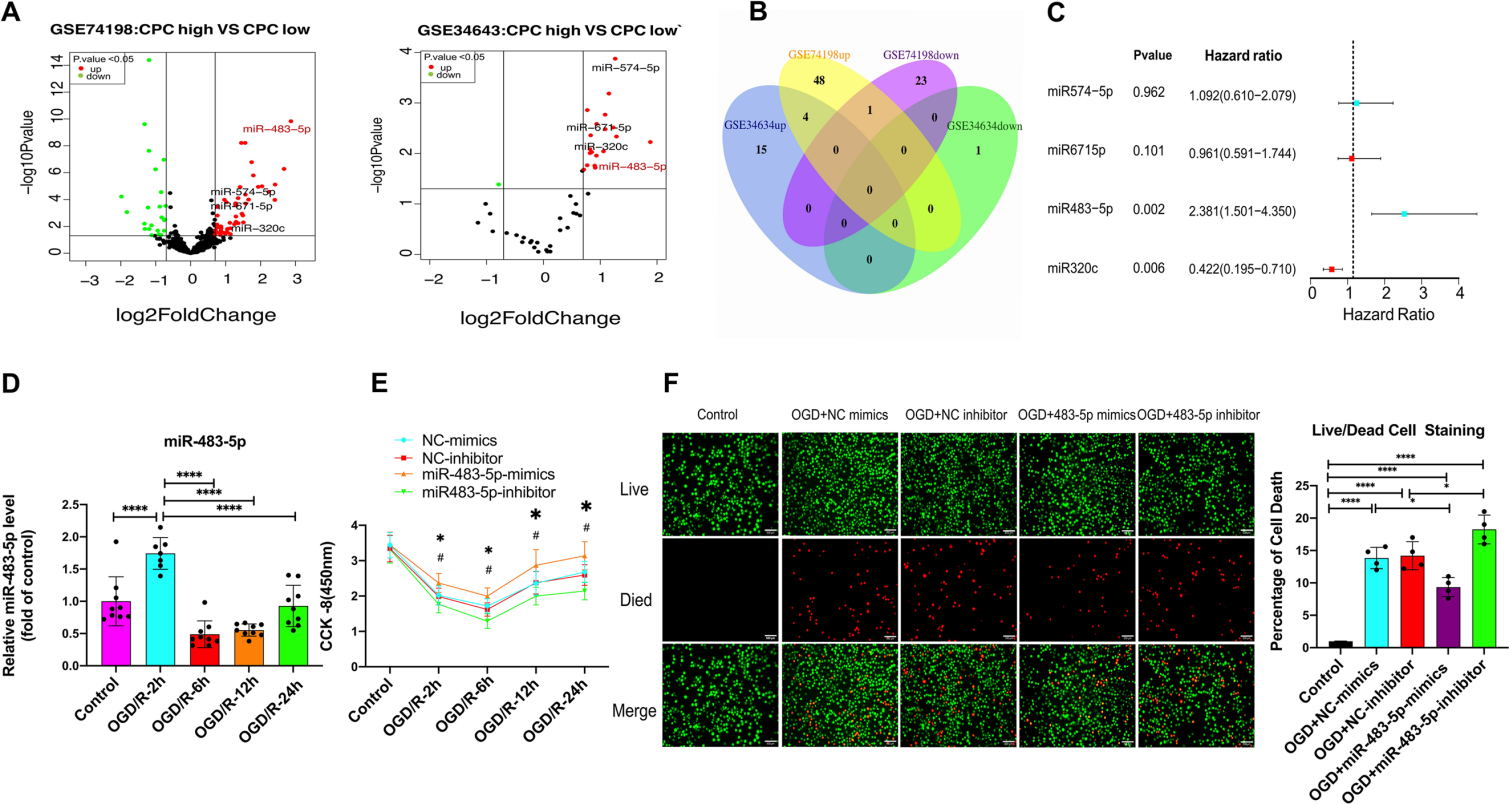
MiR-483-5p expression was associated with neurological prognosis in cardiopulmonary resuscitation patients, and upregulation of miR-483-5p diminished cell ischemia‒reperfusion injury. **A** Volcano plot of the miRNAs differentially expressed between the CPC-high and CPC-low groups. Green, decreased expression; red, increased expression. **B** Venn diagram of miRNAs. **C** Forest plots of logistic regression analysis results. **D** Expression of miR-483-5p was examined in control and 2 h, 6 h, 12 h, and 24 h after OGD/R in PC12 cells, *n* = 6. **E** Determination of cell viability at different time points after ischemia‒reperfusion injury. *P < 0.05, the miR-483-5 mimic group compared with the miR-NC mimic group; ^#^*P* < 0.05, the miR-483-5p inhibitor group compared with the miR-NC inhibitor group, *n* = 4. **F** Live/Dead cell staining assay for distinguishing live and dead cells. Live cells fluorescent green, and dead cells fluorescent red, Scale bar: 500 μm, *n* = 4. The data are expressed as the mean ± SD, **P < 0.01, ****P < 0.001

Research has demonstrated that miRNAs play active roles in CA showing predicting capacity and potential therapeutic applications (Stammet 2017). To identify the miRNAs involved in regulating neurological prognosis, the GSE74198 and GSE343634 datasets containing miRNA expression profiles of the peripheral blood of patients after cardiac arrest were downloaded from the GEO databases, and data on differentially expressed miRNAs between patients with good and poor neurological prognosis were obtained. We found that four miRNAs (miR-574-5p, miR-671-5p, miR-320c, and miR-483-5p) were identified as the differentially expressed miRNAs that were upregulated in both datasets (Fig. 1A, B). The results of logistic regression analysis demonstrate that miR-483-5p exerted the strongest effect as an independent predictor of neurological prognosis (*P* = 0.002, hazard ratio = 2.381, Fig. 1C). Then, we measured the expression of miR-483-5p after OGD/R in PC12 cells. The level of miR-483-5p increased significantly at 2 h after ischemia‒reperfusion but decreased at 6–12 h after reperfusion. The expression level gradually returned to normal after OGD/R for 24 h (Fig. 1D). A cell viability assessment showed that overexpression of miR-483-5p (Fig. S1) reduced the extent of cell injury, suggesting that miR-483-5p mimics exert a protective effect against ischemia‒reperfusion injury (Fig. 1E). In contrast, inhibition of miR-483-5p expression led to the opposite effect (Fig. 1E). These protective effects were also confirmed by live/dead cell staining (Fig. 1F).

### miR-483-5p Attenuates Ischemia–Reperfusion-Induced Neuronal Apoptosis

**Fig. 2:**
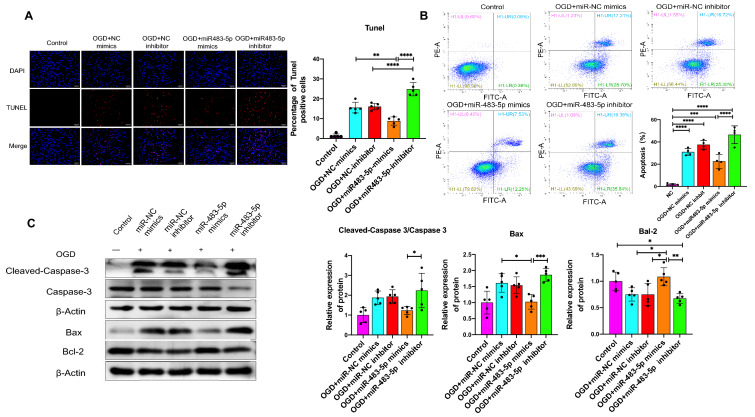
Effects of miR-483-5p on OGD/R-induced PC12 cells apoptosis. **A** TUNEL staining was performed to determine the apoptosis rate. Blue fluorescence: nucleus, red fluorescence: apoptotic cells; Scale bar: 500 μm, *n* = 6. **B** Flow cytometry results of a Annexin V-FITC-PI assay with PC12 cells after OGD/R, *n* = 4. **C** Western blot analyses of apoptosis-related proteins. β-Actin was used as the loading control. The data are expressed as the mean ± SD, *n* = 5, *P < 0.05, and **P < 0.01, ***P < 0.001, and ****P < 0.0001

As demonstrated by the CCK-8 assay and live/dead cell staining assay, miR-483-5p mimics significantly increased cell viability and decreased mortality in PC12 cells. To further characterize the function of miR-483-5p, we explored the effectiveness of miR-483-5p in regulating apoptotic cell death, which plays a decisive role in determining the prognosis of neurological function after cardiopulmonary resuscitation (Wang and Bennett 2012). The TUNEL assay revealed that overexpression of miRNA-483-5p attenuated hypoxia-induced PC12 cell apoptosis; a miR-483-5p inhibitor reversed the effect of the miRNA on apoptosis (Fig. 2A). The apoptosis rate observed by TUNEL was consistent with that observed by flow cytometry assay (Fig. 2B). Cleaved caspase 3, the active form of Caspase 3, is a marker of apoptosis. Bax is a proapoptotic protein related to the mitochondrial apoptosis pathway. As shown by western blot assay, the protein levels of cleaved caspase 3 and Bax were increased after OGD/R, and the administration of miR-483-5p mimics significantly decreased their levels. After transfection with miR-483-5 mimics, Bcl-2 expression was increased after OGD/R, while Bax and cleaved caspase 3 expression were decreased (Fig. 2C). However, a miR-483-5 inhibitor led to the opposite effect. These results demonstrate that the mitochondrial apoptosis pathway was involved in OGD/R-induced PC12 cell injury and that overexpression of miR-483-5p reduced the apoptosis rate triggered by OGD/R.

### miR-483-5-p Alleviates PC2 Cell Ischemia‒Reperfusion Injury by Promoting Mitochondrial Biogenesis and Decreasing Oxidative Stress

**Fig. 3:**
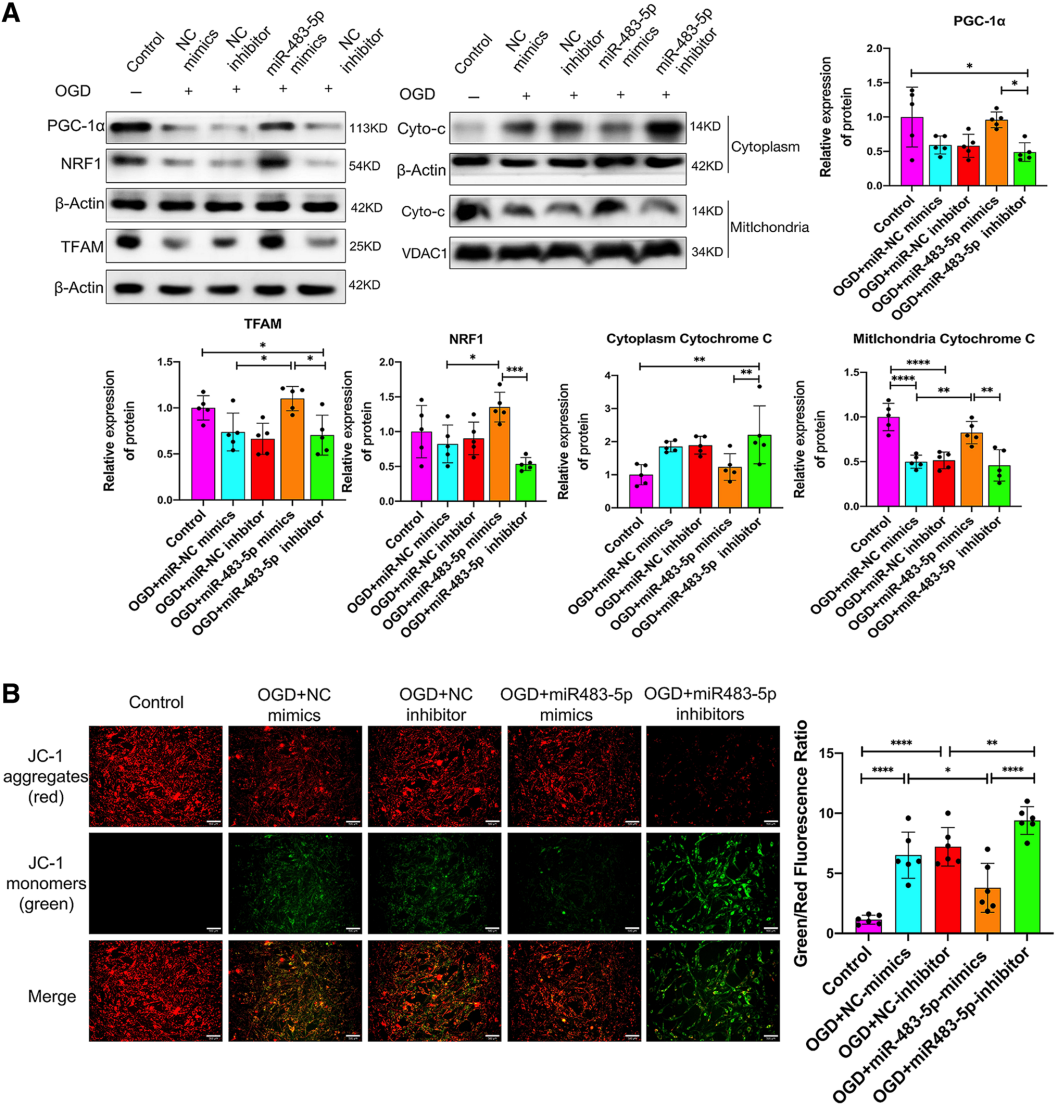

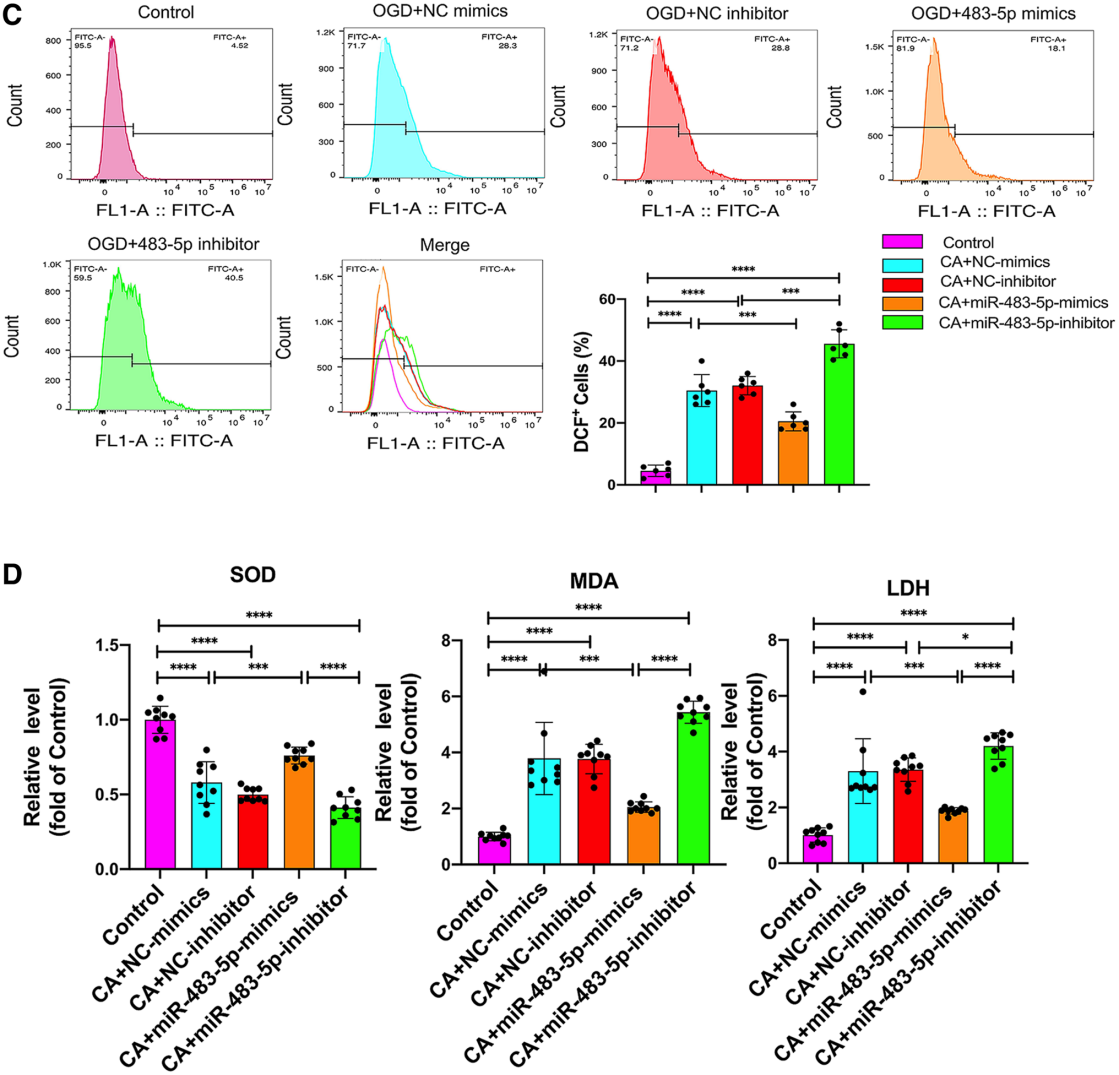
MiR-483-5-p alleviates ischemia–reperfusion mitochondrial injury in PC1 2 cells by promoting mitochondrial biogenesis and decreasing oxidative stress. **A** Western blot analyses of mitochondrial biogenesis-related proteins and the level of cytochrome c were measured both in mitochondria and the cytosol, *n* = 5. **B** Detection of mitochondrial transmembrane potential by JC-1. Red fluorescence: normal mitochondrial membrane potential, as indicated by the formation of JC-1 dimers; green fluorescence: depolarized membrane potential, as indicated by an increase the number of JC-1 monomers, Scale bar: 500 μm, *n* = 6. **C** Reactive oxygen species (ROS) were detected by flow cytometry and analyzed, *n* = 6. **D** SOD activity and MDA and LDH levels in PC12 cells after OGD/R. *n* = 3, technical replicates = 2, *P < 0.05, **P < 0.01, ***P < 0.001, ****P < 0.0001

Mitochondrial biogenesis is closely associated with oxidative stress and mitochondrial damage and promoting mitochondrial biogenesis exerts a neuroprotective effect after cerebral ischemia–reperfusion injury (Cardanho-Ramos and Morais 2021). Proliferator-activated receptor-γ coactivator α (PGC-1α) is a transcriptional coactivator and a master regulator of mitochondrial biogenesis, including mitochondrial biogenesis in the nervous system (Besseiche et al. 2015). PGC-1α and its downstream signaling intermediates, such as nuclear respiratory factor 1 (NRF1) and mitochondrial transcription factor A (TFAM), are likely important to normal mitochondrial regulation after ischemia‒reperfusion injury (Li et al. 2016). To determine the effect of miR-483-5p on mitochondrial biogenesis after OGD/R, we assessed the protein levels of PGC-1α, NRF1, and TFAM by western blotting. Additionally, we measured the levels of cytochrome c in the cytoplasm and mitochondria. The results revealed that mitochondrial biogenesis was inhibited with the levels of PGC-1α, NRF1, and TFAM decreased after OGD/R, and cytochrome c was released from the mitochondria. In the miR-483-5p mimic group, PGC-1α, NRF1 and TFAM protein levels were significantly increased, and a low level of cytoplasmic cytochrome c was detected (Fig. 3A). Consistently, the decline in mitochondrial membrane potential induced by OGD/R was reversed by the upregulation of miR-483-5p (Fig. 3B). We also detected ROS levels via flow cytometry and measured SOD activity, MDA levels, and LDH levels. Overexpression of miR-483-5p attenuated OGD/R-induced damage by inhibiting ROS generation in PC12 cells. It also inhibited the production of MDA and increased the activity of SOD (Fig. 3C, D).

### MiR-483-5p Directly Targets TNFSF8 to Regulate Mitochondrial Biogenesis and Inhibit Apoptosis After Ischemia–Reperfusion

**Fig. 4:**
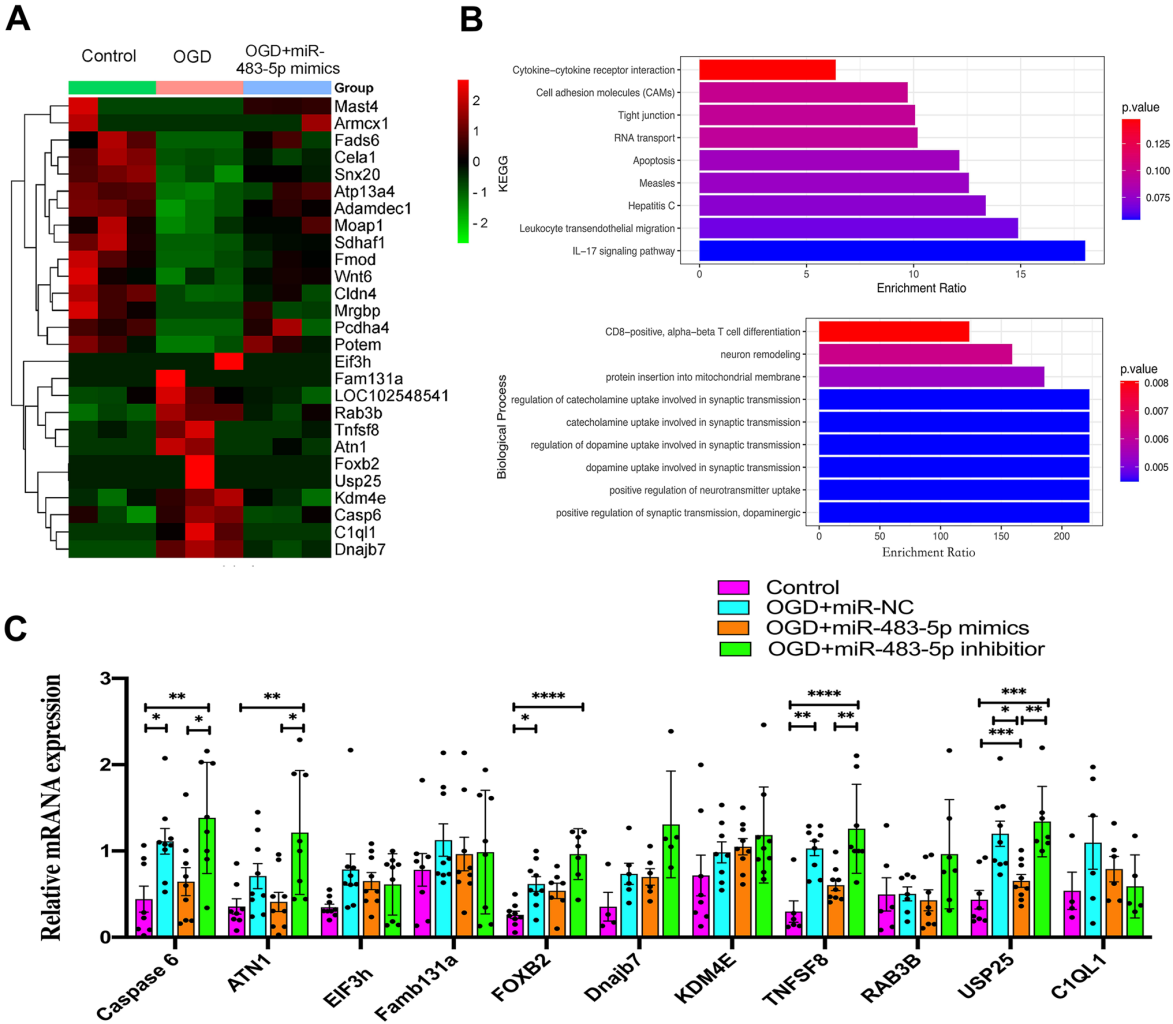

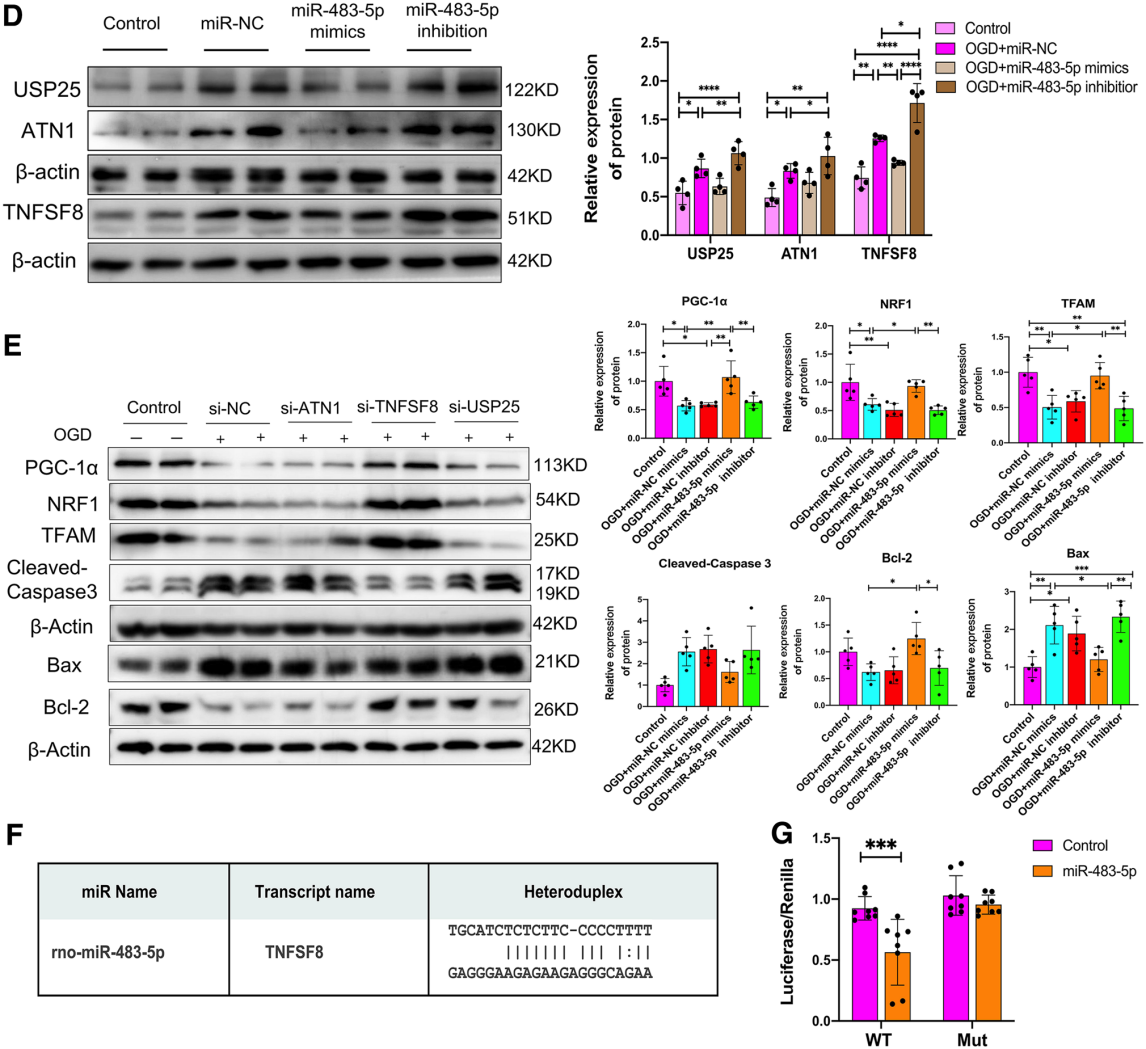
MiR-483-5p directly targets TNFSF8 to promote mitochondrial biogenesis and inhibits apoptosis after ischemia‒reperfusion. **A** Heatmap of RNA sequencing in PC12 cells (11 genes were identified as candidate target genes). **B** GO enrichment analysis and KEGG enrichment analysis with the 11 identified genes. **C** Reference gene validation in PC12 cells via qPCR. Four genes that showed significant differences were identified. **D** Validation of potential target genes as determined by western blotting, *n* = 4. **E** Effect of potential target genes involved in apoptosis and mitochondrial biogenesis in PC12 cells as determined by western blotting, *n* = 5. **F**, **G** Binding site prediction and dual-luciferase reporter assays were performed to investigate the relationship between miR-483-5p and TNFSF8, *n* = 8. *P < 0.05, **P < 0.01, ***P < 0.001, and ****P < 0.0001

miRNAs generally bind to the 3′-UTR of their target mRNAs and suppress protein production by destabilizing the mRNA, abrogating their translation (Cannell et al. 2008). RNA sequencing was performed to investigate the potential mechanism of miR-483-5p effects on OGD/R-induced cellular injury. Eleven candidate genes (C1ql1, Rab3b, Dnajb7, KDM4E, Foxb2, Eif3h, Fam131a, Tnfsf8, Usp25, Caspase 6, and Atn1) were identified as the most likely target genes of miR-483-5p that contribute to resistance against ischemia–reperfusion injury. Their mRNA expression levels increased after OGD/R but decreased after transfection with miR-483-5p mimics in PC12 cells (Fig. 4A). GO and KEGG analyses indicated that these genes were enriched in “positive regulation of synaptic transmission, dopaminergic,” “positive regulation of neurotransmitter uptake,” and “neuron remodeling,” “apoptosis,” and “RNA transport” (Fig. 4B). Then, validation of reference genes for qPCR assays was performed, and the expression of four mRNAs (Usp25, Atn1, Tnfsf8, and Caspase 6) was found to be increased after OGD/R and was decreased in PC12 cells transfected with miR-483-5p mimics (*P* < 0.05). The opposite trend was observed in the miR-483-5p inhibitor group (Fig. 4C). As miR-483-5p possibly binds sites to the 3′-UTR of three genes, USP25, ATN1, and TNFSF8 (Fig. S2), but not Caspase 6, a potential indirect interaction between miR-483-5p and Caspase 6 was inferred. Western blot analysis indicated a consistent correlation between the protein and mRNA levels of ATN1, USP25, and TNFSF8 (Fig. 4D). Using short interfering RNAs (siRNAs), we silenced the protein expression of the ATN1, USP25, and TNFSF8 (Figure S3). Next, we investigated the effects of protein expression silencing on apoptosis and mitochondrial biogenesis after OGD/R via western blot analysis. The inhibition of TNFSF8 significantly reduced the protein levels of cleaved caspase 3 and Bax while promoting the expression of mitochondrial biogenesis-related proteins, such as PGC-1α, NRF1, and TFAM (Fig. 4E). These findings suggest that miR-483-5p may reduce OGD/R damage by targeting TNFSF8. Finally, TNFSF8 was confirmed to be a direct target of miR-483-5p, as indicated via a dual-luciferase reporter assay (Fig. 4F).

### miR-483-5p Promotes Mitochondrial Biogenesis and Inhibits Apoptosis by Regulating the AMPK/JNK Pathway after Ischemia‒Reperfusion

**Fig. 5:**
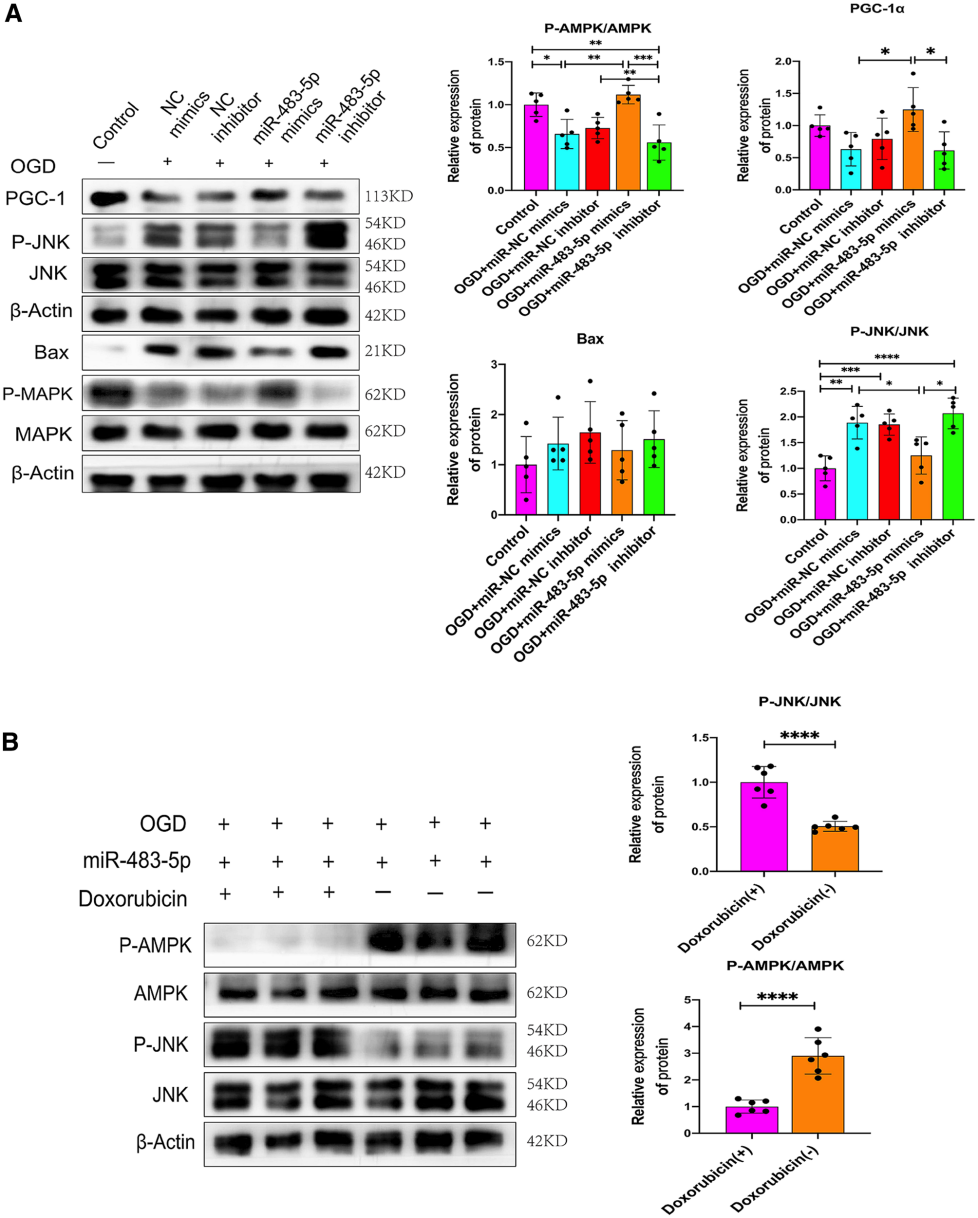

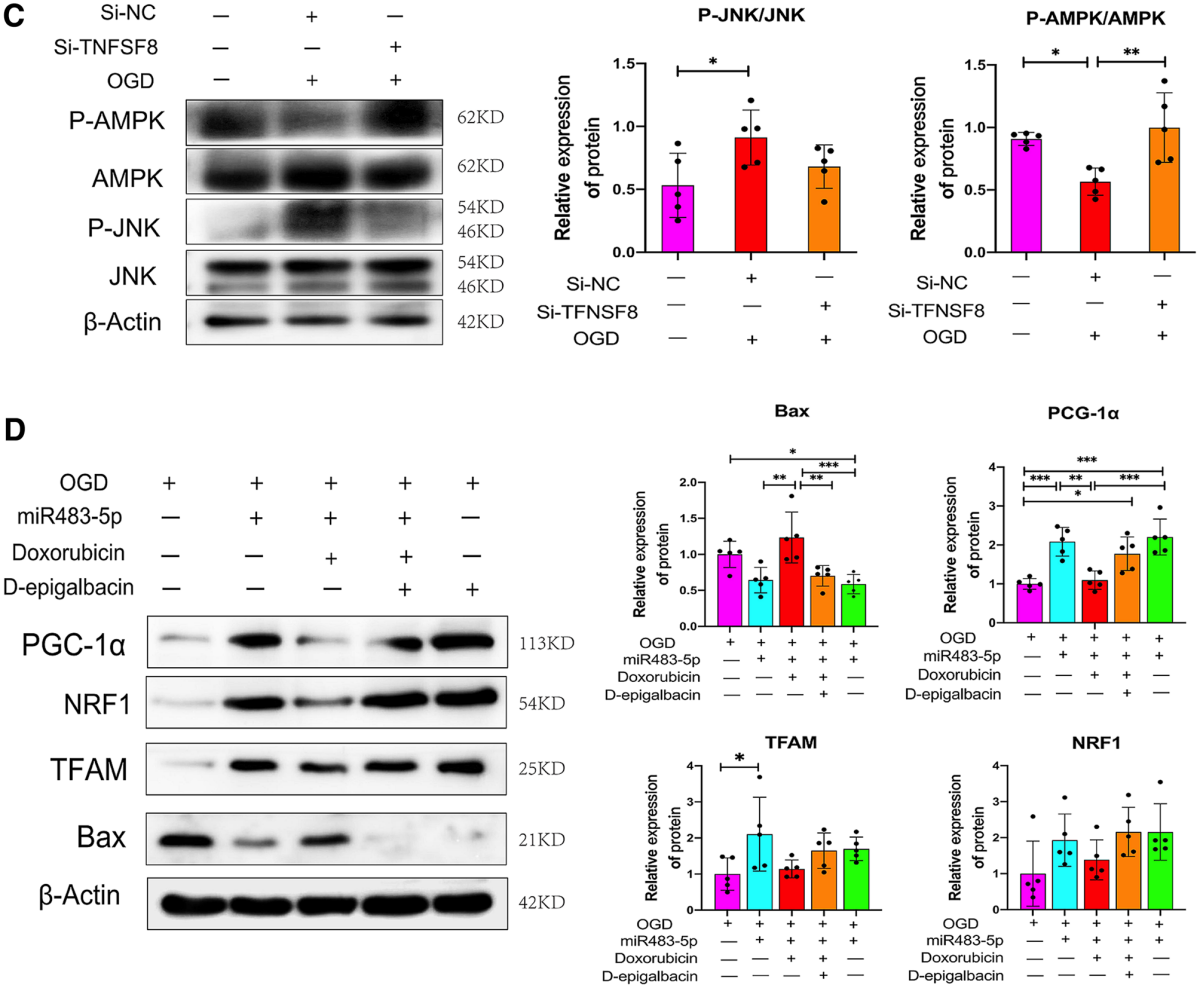
MiR-483-5p determines the activity of the TNFSF8/AMPK/JNK pathway. **A** MiR-483-5p enhanced the activity of AMPK while decreasing JNK pathway activity after OGD/R, as confirmed by Western blot assay, *n* = 5. **B** AMPK phosphorylation inhibits JNK phosphorylation after ischemia–reperfusion injury, *n* = 5. **C** Inhibition of TNFSF8 expression decreased AMPK activity while increasing JNK activity, *n* = 5. **D** Both AMPK activity inhibition and JNK activation inhibition counteracted the protective effect of miR-483-5p. Inhibition of JNK activity exerted a protective effect, *n* = 5. The data are expressed as the mean ± SD. *P < 0.05, **P < 0.01, ***P < 0.001, and ****P < 0.0001. Doxorubicin, an AMPK activator inhibitor; d-epigabacin, a JNK activator inhibitor

Researchers found that oxidative stress-induced mitochondrial-dependent apoptosis is associated with AMPK/JNK signaling pathway activation (Lou et al. 2021). Furthermore, previous studies have shown that AMPK regulates mitochondrial biogenesis and that the JNK pathway is critical for regulating ROS-mediated apoptosis (Thirupathi and de Souza 2017). We measured the phosphorylation levels of JNK and AMPK by western blotting to further investigate their relationship with miR-483-5p. A significant decrease in AMPK phosphorylation and PGC-1α protein levels was observed in PC12 cells after OGD/R, whereas JNK phosphorylation and Bax protein levels were markedly increased. Moreover, miR-483-5p mimics downregulated the expression of phosphorylated JNK and Bax and increased the protein levels of phosphorylated AMPK and PGC-1α (Fig. 5A). Subsequently, by the AMPK activity inhibitor doxorubicin was used, and it was found that after inhibiting the activity of AMPK, the phosphorylation level of JNK increased (Fig. 5B). As TNFSF8 is a target gene of miR-483-5p, we then explored the relationship between TNFSF8 and the AMPK/JNK pathway. By transfecting siRNA to inhibit the expression of TNFSF8 after OGD/R, the activity of AMPK increased and that of JNK decreased (Fig. 5C). In addition, we found that the protective effect of miR-483-5p were blocked when AMPK activity was inhibited and that protective effects of miR-483-5p were restored after treatment with d-epigalbacin (Fig. 5D). These results suggest that miR-483-5p plays a protective role that is mediated through the TNFSF8/AMPK/JNK pathway.

### MiR-483-5p Alleviates Brain Injury and Improves Neurological Function After Cardiac Arrest in Rats

**Fig. 6:**
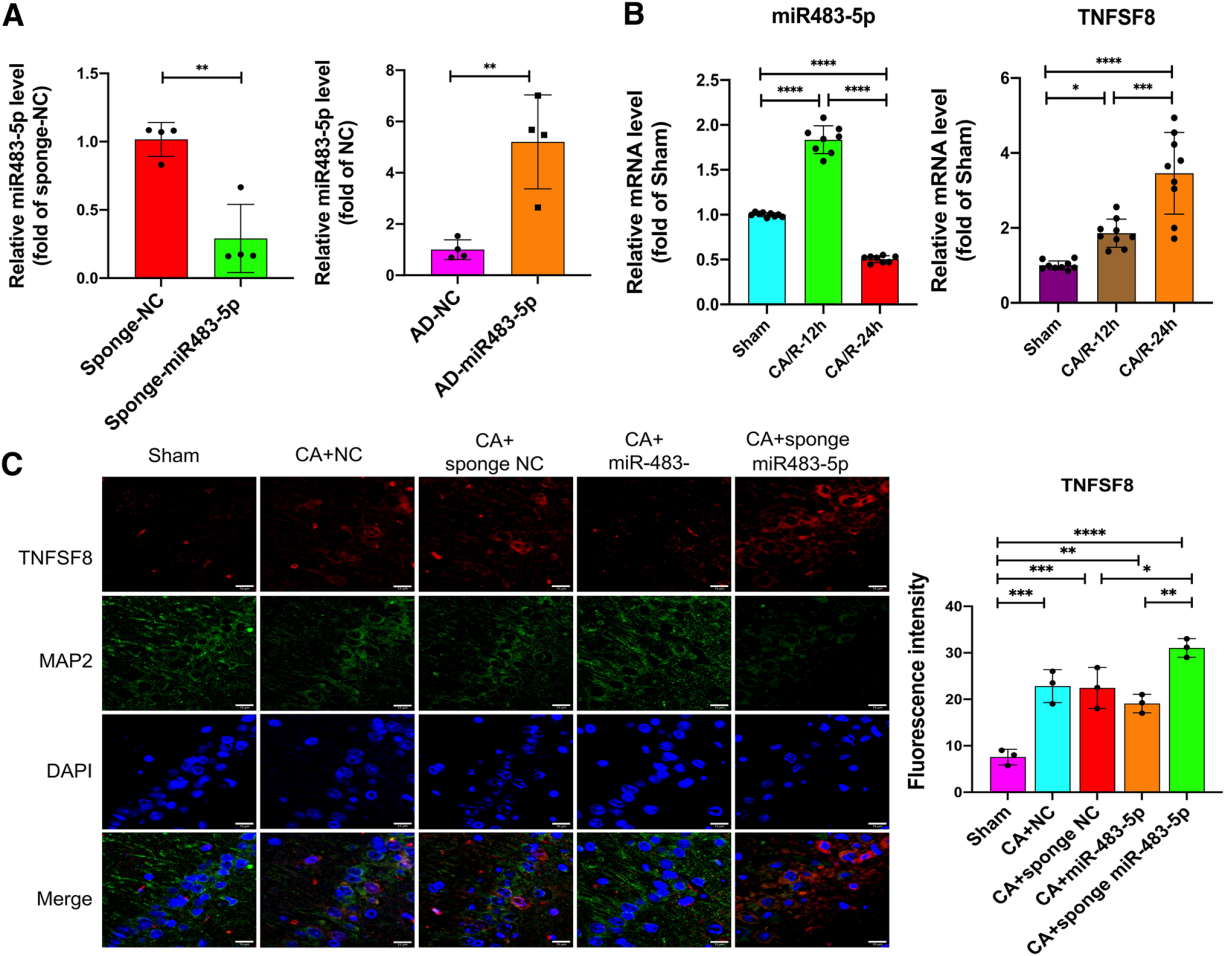

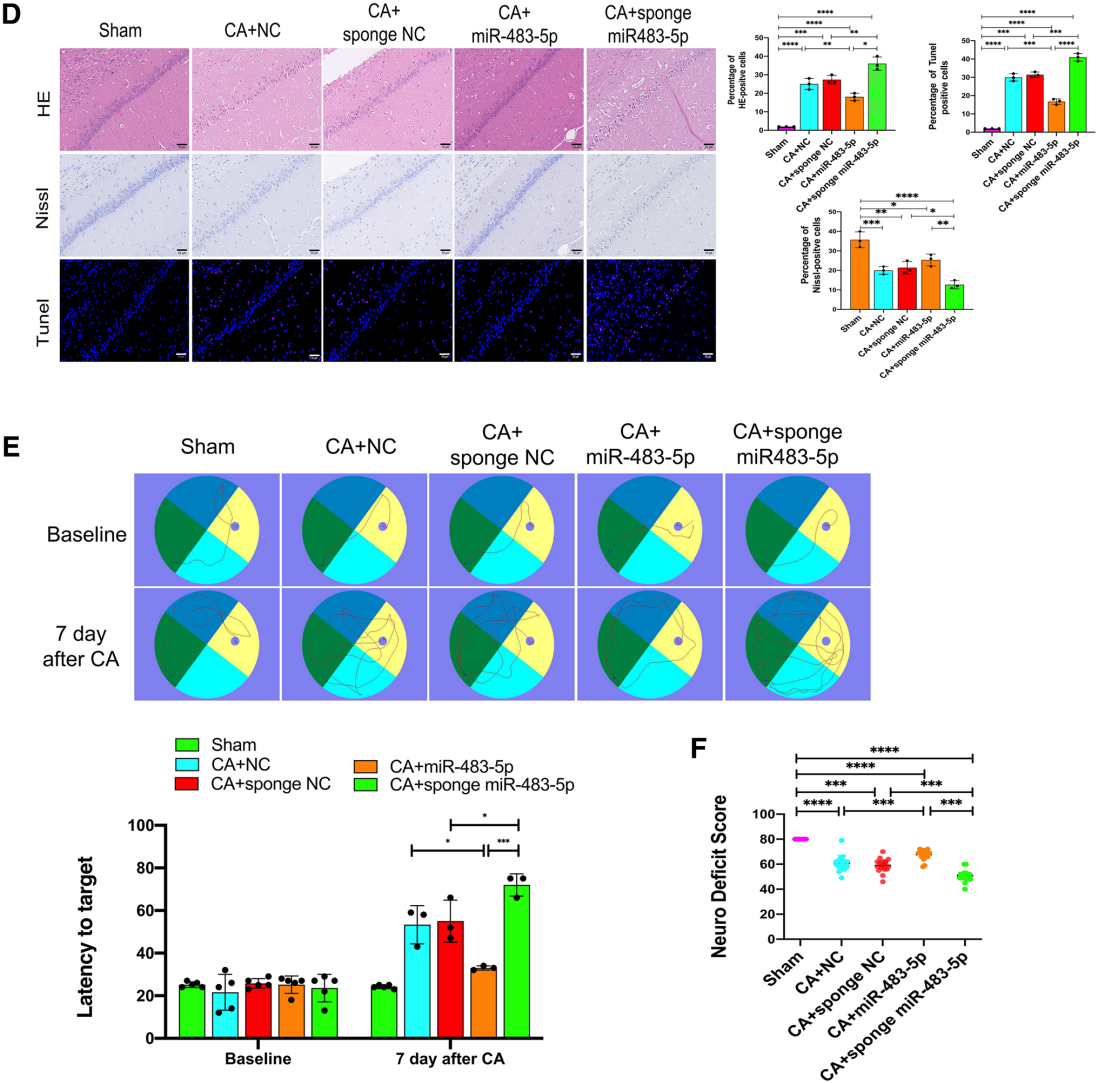
MiR-483-5p alleviates brain injury after cardiac arrest in rats. **A** Expression of adeno-associated virus carrying miR-NC, miR-483-5p, sponge miR-NC, and sponge miR 483-5p in rat hippocampus 4 weeks after transfection, as determined by qPCR, *n* = 2; technical replicates = 2. **B** Expression of miR-483-5p and tnfsf8 mRNAs in the rat hippocampus 24 h after cardiopulmonary resuscitation. **C** Immunofluorescence assay for TNFSF8 protein detection in the rat hippocampus, Scale bar: 15 μm, *n* = 3. **D** HE staining, Nissl staining, and TUNEL staining of hippocampal tissues in the CA1 region of rats from each group, Scale bar: 50 μm, *n* = 3. **E**, **F** An NDS score was obtained and a Morris water maze test was performed to assess spatial and related forms of learning and memory after cardiac arrest for 24 h, *n* = 3. The data are expressed as the mean ± SD. *P < 0.05, **P < 0.01, ***P < 0.001, and ****P < 0.0001

We established a miR-483-5p overexpression group and miR-483-5p inhibition group in rats via intracranial injection of adeno-associated viral vectors. Four weeks later, qPCR was used to determine miR-483-5p expression in each group (Fig. 6A). Furthermore, we measured the mRNA expression of TNFSF8 and miR-483-5p in the rat hippocampus after cardiopulmonary resuscitation. MiR-483-5p expression increased 12 h after cardiopulmonary resuscitation but was decreased at 24 h. TNFSF8 mRNA levels increased significantly after cardiopulmonary resuscitation (Fig. 6B). Immunofluorescence examination of TNFSF8 protein expression in the rat hippocampus was performed, and the result was consistent with that obtained for the mRNA expression. Negligible protein expression of TNFSF8 was detected in the normal rat hippocampus. In contrast, clear expression of TNFSF8 was observed on some of the hippocampal neurons of rats after cardiac arrest (Fig. 6C). Upregulating miR-483-5p inhibited the protein expression of TNFSF8, while inhibiting miR-483-5p increased the protein expression of TNFSF8 after cardiac arrest (Fig. 6C). Representative pictures showing H&E staining, Nissl staining, and TUNEL staining are displayed in Fig. 6D. HE staining revealed that hippocampal neurons in the CA1 region in the sham group were arranged in an orderly manner with normal structures, obvious nucleoli, and clear nuclei and abundant cytoplasm. Many swollen neurons with loosened structures, karyopyknosis, and vacuolar structures were observed in the miR-NC group after CA. The pathological changes in hippocampal neurons were ameliorated in the miR-483-5p group. After CA, the miR-483-5p inhibition group showed more obvious hippocampal damage than the miR-NC inhibition group. As shown by Nissl staining, the number of positive neurons in the CA1 hippocampus was significantly decreased after CA (*P* < 0.05) and was significantly increased after administration of miR-483-5p (*P* < 0.05). Neuronal apoptosis was detected by TUNEL assay, and the positive cells were stained red; miR-483-5p directly attenuated CA-induced hippocampal neuronal apoptosis in cardiac arrest. A Morris water maze test was performed and the neuropathy diabetes score (NDS) was determined to assess neurological function and memory 24 h after CA. Compared with the miR-NC group, the miR-483-5p group showed a higher NDS score (Fig. 6E). There was no difference in the baseline latency time to target for the rats in each group before CA. The miR-483-5p group rats found the target platform faster, indicating good neural function. The miR-483-5p inhibition group spent a long time finding the platform (Fig. 6F).

### miR-483-5p Improves Mitochondrial Biogenesis and Reduces Oxidative Stress-Induced Apoptosis in Rats After Cardiac Arrest

**Fig. 7:**
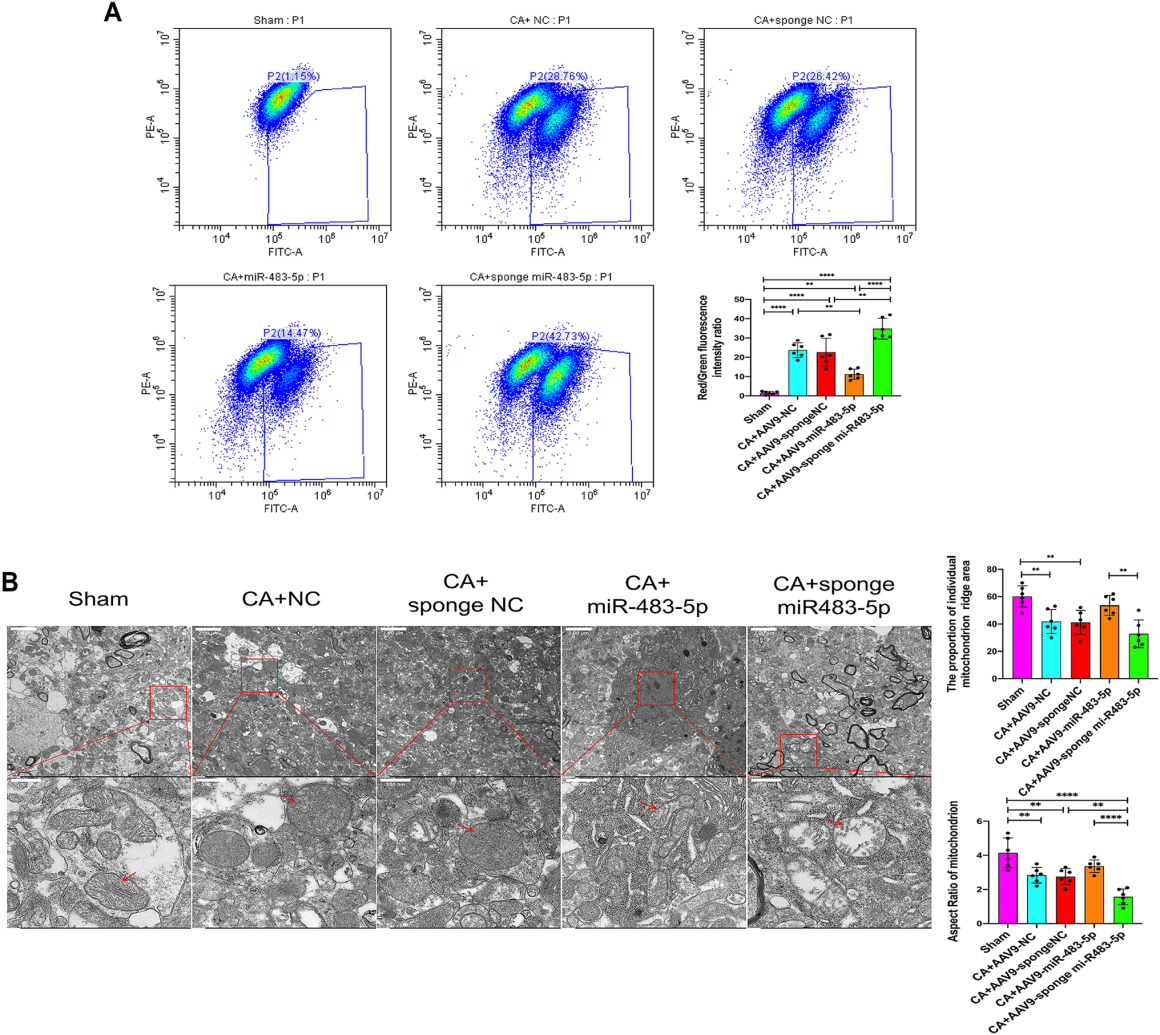

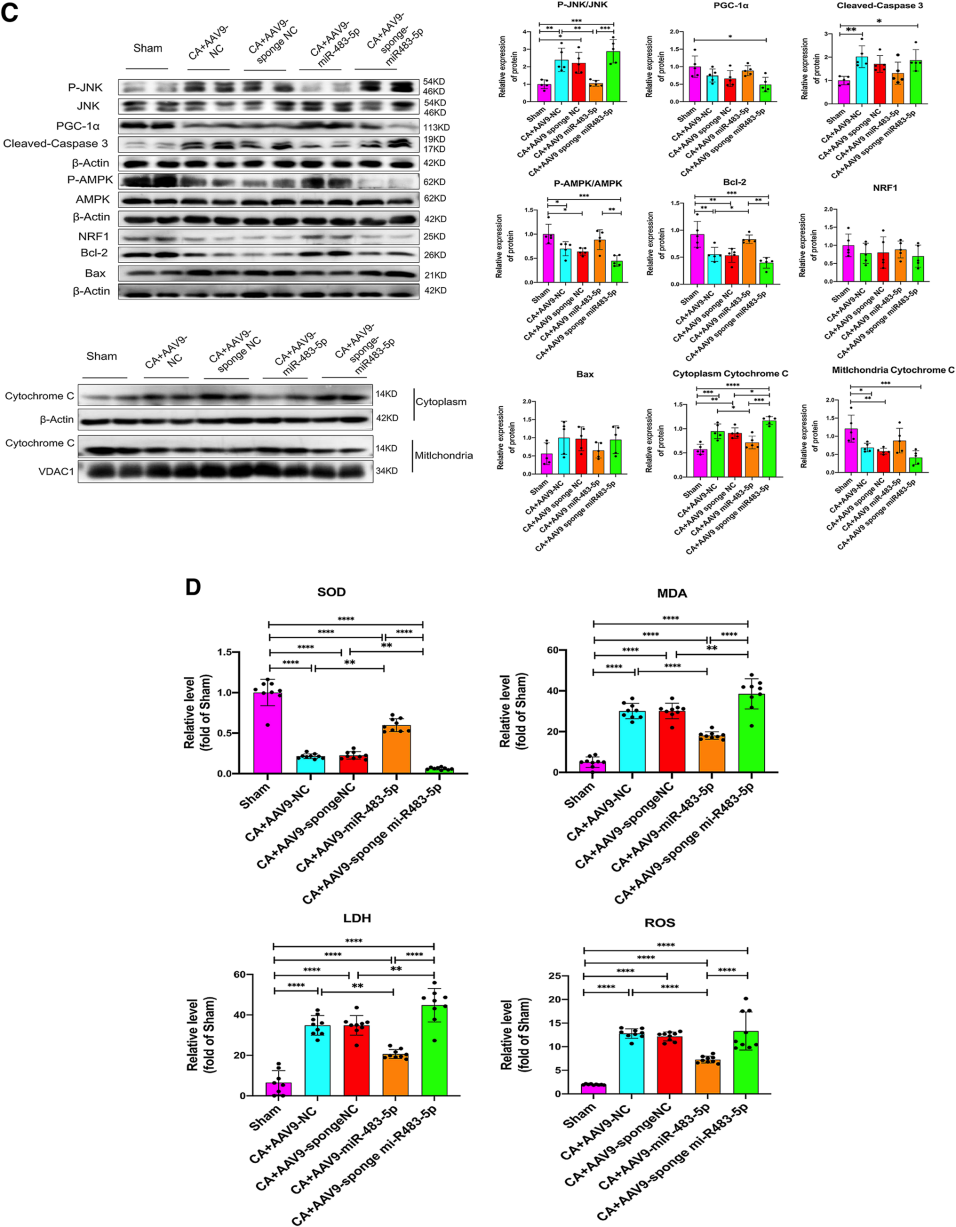
MiR-483-5p alleviates brain ischemia–reperfusion injury by inhibiting excessive mitochondrion-dependent apoptosis, promoting mitochondrial biogenesis, and decreasing oxidative stress in rats. **A** Changes in mitochondrial membrane potential was assessed by adding JC-1 to hippocampal neurons, *n* = 3; technical replicates = 2. **B** Electron microscopy revealed the morphology of mitochondria in the rat hippocampus after cardiac arrest (red arrow: mitochondrion with an intact outer membrane and dense cristae, severely impaired mitochondria with fuzzy cristae and swelling matrix (Above scale bar: 2.00 μm; below scale bar: 500 nm), *n* = 3; technical replicates = 2. **C** The expression of proteins related to mitochondrial biogenesis and apoptosis in rat hippocampal tissues from each group was measured by Western blotting, *n* = 5. **D** SOD and MDA activity and ROS and LDH levels were measured in hippocampal tissues after cardiac arrest, *n* = 3; technical replicates = 3. The data are expressed as the mean ± SD. *P < 0.05, **P < 0.01, ***P < 0.001, and ****P < 0.0001

A flow cytometry-based assay with the cationic dye JC-1 was performed to measure mitochondrial membrane potential (MMP) in hippocampal tissue. The MMP was quantified by measuring the green-shifted monomers in the FL-1 channel and the redshifted JC-1 aggregates in the FL-2 channel. The results showed that the MMP of hippocampal tissue decreased, as indicated by enhanced green fluorescence after CA, while miR-483-5p inhibited the generation of green fluorescence (Fig. 7A). The mitochondria exhibited an intact outer membrane and dense cristae in the sham group; however, the mitochondria were clearly impaired, as indicated by fuzzy cristae and matrix swelling, after CA in the miR-NC and miR-NC inhibition groups. Overexpression of mR483-5p improved the mitochondrial morphology, that is, the mitochondrial cristae were distinct and little swelling in the matrix was observed compared with those in the model group. Moreover, the mitochondrial cristae and matrix in the miR-483-5p inhibition group were more severely damaged than those in the miR-NC inhibition group (Fig. 7B). Performing western blot assays, we evaluated the effects of miR-483-5p on mitochondrial biogenesis and apoptosis after CA. The results showed that the protein expression levels of p-AMPK, PGC-1α, NRF1, Bal-2, and TFAM were significantly decreased in the CA group compared with those in the sham group (*P* < 0.05). In the miR-483-5p group after CA, the protein expression levels of p-AMPK, PGC-1α, NRF1, Bal-2, and TFAM were markedly increased compared with those in the miR-NC group (miR-483-5p inhibition reversed these effects, *P* < 0.05). Upregulated expression of miR-483-5p suppressed the expression of the proapoptotic proteins Bax and cleaved caspase 3 following cardiopulmonary resuscitation while increasing the expression of the antiapoptotic protein Bcl-2 (*P* < 0.05). The sponging miR-483-5p led to the opposite result: apoptosis (Fig. 7C). Additionally, we determined the oxidative stress level in the rat hippocampus for each rat group. Following cardiac arrest, the oxidative stress levels in the rat hippocampus increased significantly with increased LDH levels; however, MDA and SOD activity and LDH levels were reduced in the miR-483-5p group (Fig. 7D).

## Discussion

**Fig. 8:**
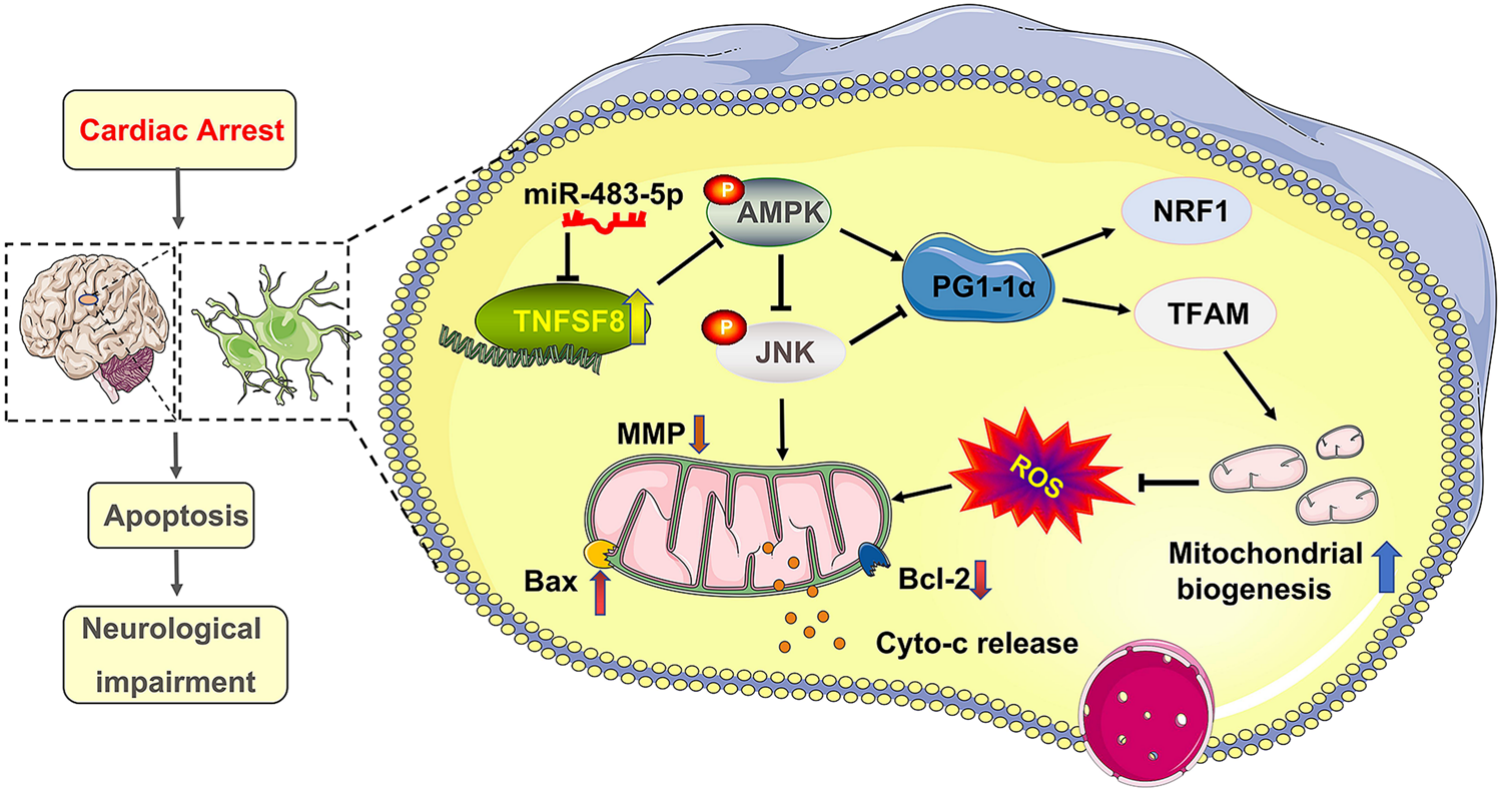
Hypothetical mechanism through which miR-483-5p attenuates ischemia‒reperfusion injury after cardiopulmonary resuscitation. miR-483-5p promotes mitochondrial biogenesis and reduces apoptosis via the TNFSF8/AMPK/JNK pathway

Delayed brain mitochondrial dysfunction during ischemia‒reperfusion is the main reason for the severity of neurological injury (Kohlhauer et al. 2021). In the present study, our results revealed a close relationship between mitochondrial function and brain function after cardiac arrest. Indeed, mitochondrial homeostasis is necessary to maintain neuronal homeostasis as neurons have a high energy demand. In addition, mitochondria are the main sources of reactive oxygen species. Our study showed that miR-483-5p targets TNFSF8 to protect against neurological impairment by promoting mitochondrial biogenesis and reducing oxidative stress damage (Fig. 8).

Postcardiac arrest brain injury (PCABI) caused by subsequent brain reperfusion following resuscitation is the leading cause of mortality and long-term disability. MicroRNAs have recently emerged as potential biomarkers of neurological prognosis, and they are also therapeutic targets in cardiac arrest diseases (Devaux et al. 2015). In this study, we focused on identifying the alteration of miRNAs and exploring their possible mechanisms after cardiac arrest. The logistic regression analysis revealed a strong association between miR-483-5p expression and neurological prognosis for CA patients (Fig. 1C). This finding was consistent with prior research showing that miR-483-5p was closely related to neurological disease. Nagaraj et al. found that miR-483-5p was the most abundant miRNA in blood plasma, specifically in prodromal and early-stage Alzheimer’s disease (AD) patients, and upregulation of miR-483-5p decreased phosphorylation of neuronal microtubule-associated protein Tau, suggesting that it plays a neuroprotective role in AD pathology (Nagaraj et al. 2021). Expression of miR-483-5p promotes insulin-like growth factor-II (IGF-II) transcription, which is associated with neurogenesis and differentiation (Vazquez et al. 2013; Zhu et al. 2019).

To date, the role played by miR-483-5p in ischemic disease remains uncertain. Our study found that miR-483-5p exerts a protective effect by reducing ischemia‒reperfusion injury in PC12 cells and improving neurological outcomes after CA in rats. Then, we investigated the potential mechanism of miR-483-5p. After ischemia‒reperfusion, miR-483-5p promoted mitochondrial biogenesis by upregulating the protein expression of PGC-1α, inhibited the production of ROS, and reduced the mitochondrion-dependent apoptosis rate both in vivo and in vitro. Furthermore, miR-483-5p significantly increased the phosphorylation of AMPK while decreasing the phosphorylation of JNK. These data showed that miR-483-5p treatment exerted a strong neuroprotective effect after cardiac arrest.

Multiple mitochondria-related mechanisms have been proposed to play essential roles in brain ischemia–reperfusion (I-R) injury. Mitochondrial dysfunction following ischemic stroke results in the loss of mitochondrial membrane potential, generation of ROS, and activation of intrinsic apoptosis (Li et al. 2018). In animal models, some therapeutic interventions targeting mitochondrial dysfunction have proven effective for brain injury following cardiopulmonary resuscitation. For example, palmitic acid therapy after CA inhibited neuroinflammation and mitochondrial dysfunction, improving neurological function (Wu et al. 2021). PD98059 protected cerebral cortex mitochondrial structure, increased survival rates and neurological deficit scores, and reduced the rate of mitochondrial permeability transition pore opening (Zheng et al. 2020). Therefore, mitochondria have become increasingly important targets for therapeutic interventions in cardiac arrest-induced brain injury.

Increasing evidence shows that the promotion of mitochondrial biogenesis protects neurons against mitochondrial insults and improves neurological outcomes after cardiac arrest in rats (Pan et al. 2014; Wang et al. 2016). PGC-1α has been reported to be a major regulator of mitochondrial biogenesis by inducing biogenesis via the activation of different transcription factors, including nuclear respiratory factor 1 (NRF 1) and mitochondrial transcriptional factor A (TFAM) (Piccinin et al. 2019). Increased PGC-1α expression was associated with a reduction in ROS production and a subsequent increase in mitochondrial biogenesis (Sharma et al. 2015). Evidence has also shown that increased mitochondrial ROS downregulated mitochondrial biogenesis (Chevtzoff et al. 2010), while upregulating the expression of PGC-1α, increasing the expression of components of the mitochondrial ROS defense system, such as glutathione peroxidase or SOD, in neural cells, thus protecting neural cells from oxidative stress-mediated death (St-Pierre et al. 2006). Our data showed that the PGC-1α, NRF 1, and TFAM protein expression levels were increased in the rat hippocampi overexpressing miR-483-5p after ROSC, and this outcome was accompanied by an excellent neurological prognosis. This finding indicates that miR-483-5p induced mitochondrial biogenesis and promoted recovery from I-R brain injury. Notably, miR-483-5p has been shown to regulate mitochondrial dynamics in other contexts. Fan et al. and Tian et al. showed that miR-483-5p attenuated mitochondrial fission and cisplatin sensitivity and inhibited apoptosis in tongue squamous cell carcinoma (Fan et al. 2015; Tian et al. 2019a, b). Moreover, miR-483-5p promoted the expression of PPAR-γ, which participates in the regulation of mitochondrial biogenesis (Wang et al. 2022). Our results demonstrate that miR-483-5p affects brain injury after cardiac arrest by regulating mitochondrial biogenesis.

TNFSF8 functions through its interaction with CD30 and is reported to be a death ligand that induces the apoptosis and proinflammatory factor production (Ruggeri et al. 2002; Tur et al. 2009). Although TFNSF8 is rarely expressed in normal tissues and cells except when the immune system is activated (Abdalla et al. 2004), the expression of TNFSF8 has been widely detected in many tissues and cells that are under stress conditions. TNFSF8 was expressed in the cardiac myocytes of mice with myocarditis, and TNFSF8 was found to be moderately or highly abundant on ventricular myocytes after treatment with IFN-γ (Seko et al. 1999). Additionally, TNFSF8 has been observed in thyroid epithelial (follicular) cells, yolk sac carcinoma, and embryonal carcinoma (Pera et al. 1997; Ruggeri et al. 2002). Our study confirmed that TNFSF8 is expressed in hippocampal neurons. Although protein expression was relatively low in normal neurons, TNFSF8 protein expression was significantly increased and caused damage in hippocampal neurons after cardiac arrest. TNFSF8 was detected on PC12 cells. Furthermore, we demonstrate that TNFSF8 was the target gene of miR-483-5p and that inhibiting the expression of TNFSF8 played a neuroprotective role after cardiac arrest. The inhibition of TNFSF8 expression alleviated mitochondrial injury and induced mitochondrial biogenesis.

AMP-activated protein kinase (AMPK) is a highly conserved serine/threonine protein kinase and a key regulator of energy homeostasis and cell survival under inflammatory and oxidative stress conditions (Sun et al. 2013). A study documented that AMPK inactivation or activation due to oxidative stress was involved in ischemic brain damage (He et al. 2020a, b). Activated AMPK can be measured to monitor mitochondrial function because it regulates the expression of peroxisome proliferator-activated receptor-γ coactivator (PGC)-1α, attenuating neuronal injury after ischemia‒reperfusion (Tian et al. 2019a, b). Jun N-terminal kinase (JNK) activation induced ROS-modified mitochondrial fission and increased the mitochondrion-dependent apoptosis rate (Chen et al. 2021; Zhang et al. 2020a, b). Our results show that upregulating TNFSF8 inhibited the activation of AMPK while increasing the activity of JNK. JNK phosphorylation was suppressed in cells treated with the AMPK activation inhibitor doxorubicin. This finding indicated that JNK may be a downstream target of AMPK. The harmful effect of TNFSF8 was offset by treatment with the JNK activation inhibitor d-epigalbacin. This outcome suggested that TNFSF8 may function through the AMPK/JNK pathway. Some studies have shown that the AMPK/JNK pathway is related to the regulation of ROS generation and apoptosis. Lou et al. observed that inhibiting oxidative stress-induced mitochondrion-dependent apoptosis through the AMPK/JNK signaling pathway enhanced cardiac function (Lou et al. 2021). Chen et al. showed that metformin attenuated cadmium-induced neuronal apoptosis in vitro by blocking the AMPK/JNK signaling pathway (Chen et al. 2020). These results were consistent with those in our study. We found that when AMPK and JNK activity were both inhibited, the protein expression of PGC-1a, NRF1, and TFAM remained high, suggesting that the potential regulatory mechanisms between AMPK/JNK and the PGC-1α pathway are complex. To some extent, inhibition of JNK activity promoted PGC-1α protein expression. Zheng et al. reported that inhibition of JNK indirectly increased PGC-1α expression by upregulating PPAR-α (Zheng et al. 2017).

Our study was exploratory and had some limitations. The study showed that miR-483-5p suppressed the expression of Caspase 6 in PC12 cells. Caspase 6 is related to cognitive impairment and inflammation in the brain (Flores et al. 2022). However, Caspase 6 is not the direct target of miR-483-5p. Moreover, miR-483-5p reduced the expression of ATN1 and USP25. ATN1 mutation has been associated with neurodevelopment. Overexpression of USP25 resulted in microglial activation and induced synaptic and cognitive deficits (Zheng et al. 2021). Although ATN1 and USP25 carry potential binding sites for miR-483-5p, no further verification was performed. These results suggest that the neuroprotective mechanisms of miR-483-5p are multifaceted, and more research is needed.

## Conclusion

In conclusion, the present study revealed that the miR-483-5p/TNFSF8/AMPK/JNK axis was critically involved in mitochondrial dysfunction in ischemia‒reperfusion injury in vivo and in vitro. MiR-483-5p targets TNFSF8 to protect against neurological impairment by promoting mitochondrial biogenesis and reducing oxidative stress damage. These results indicate that miR-483-5p may be a new therapeutic target for improving neural function following cardiopulmonary resuscitation.

## Supplementary Information

Below is the link to the electronic supplementary material.

Supplementary file1 (DOCX 2340 KB)

## Data Availability

The data that support the findings of this study are available from the corresponding author, Xiao-Xing Liao, upon reasonable request.
