# Supplementary material for: Neuroprotective Effect of miR-483-5p Against Cardiac Arrest-Induced Mitochondrial Dysfunction Mediated Through the TNFSF8/AMPK/JNK Signaling Pathway
Source: Cell Mol Neurobiol. 2022 Oct 20;43(5):2179–202. doi: 10.1007/s10571-022-01296-3 (PMC10287582; doi:10.1007/s10571-022-01296-3)
Supplement: Supplementary file 1 — Supplementary file1 (DOCX 2340 KB) [file 10571_2022_1296_MOESM1_ESM.docx]

**Supplementary data**

Figure-S1


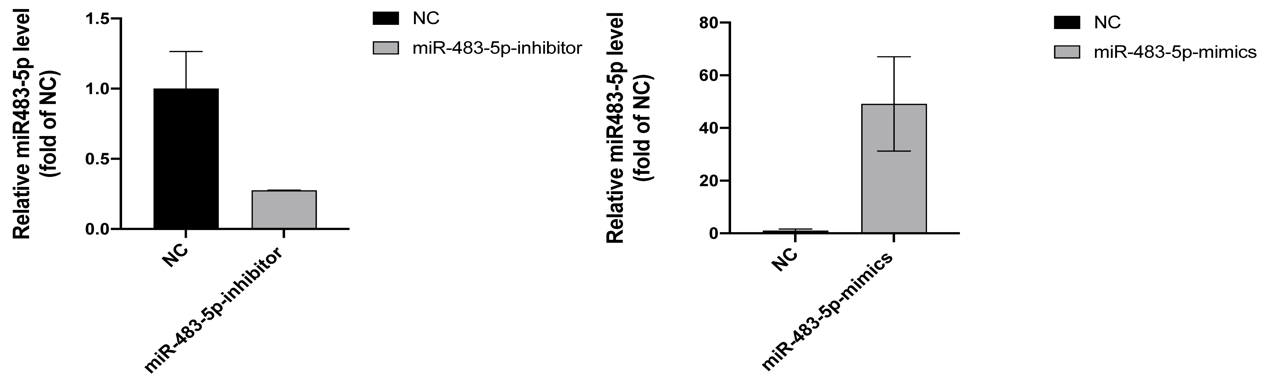


**Figure S1.** Expression of miR-483-5p after transfected with miR-NC mimics,miR-483-5p mimics, and miR-NC inhibitor, miR-483-5p inhibitor(n=3).

**Figure S2.**

**Figure S2.**  Binding sites between miR-483-5p and possible target gene ATN1， USP25.

Figure S2.

**Figure S3.** The protein expression of TNFSF8, USP25, and ATN1 in PC12 cells transfected with a small interfering RNA oligonucleotide(n=3).
